# Visualizing germination of microbiota endospores in the mammalian gut

**DOI:** 10.1080/19490976.2022.2125737

**Published:** 2022-09-29

**Authors:** Ningning Xu, Liyuan Lin, Yahui Du, Huibin Lin, Jia Song, Chaoyong Yang, Wei Wang

**Affiliations:** a Institute of Molecular Medicine, Shanghai Key Laboratory for Nucleic Acid Chemistry and Nanomedicine, Renji Hospital, Shanghai Jiao Tong University School of Medicine, Shanghai, China; b The MOE Key Laboratory of Spectrochemical Analysis and Instrumentation, Key Laboratory for Chemical Biology of Fujian Province State Key Laboratory of Physical Chemistry of Solid Surfaces, Department of Chemical Biology, College of Chemistry and Chemical Engineering, Xiamen University, Xiamen, China

**Keywords:** Bacterial endospore, germination, *in vivo* metabolic labeling, FDAA, two-color fluorescence imaging

## Abstract

Transmission of bacterial endospores between the environment and people and the following germination *in vivo* play critical roles in both the deadly infections of some bacterial pathogens and the stabilization of the commensal microbiotas in humans. Our knowledge about the germination process of different bacteria in the mammalian gut, however, is still very limited due to the lack of suitable tools to visually monitor this process. We proposed a two-step labeling strategy that can image and quantify the endospores’ germination in the recipient’s intestines. Endospores collected from donor’s gut microbiota were first labeled with fluorescein isothiocyanate and transplanted to mice via gavage. The recipient mice were then administered with Cyanine5-tagged D-amino acid to label all the viable bacteria, including the germinated endospores, in their intestines *in situ*. The germinated donor endospores could be distinguished by presenting two types of fluorescent signals simultaneously. The integrative use of cell-sorting, 16S rDNA sequencing, and fluorescence *in situ* hybridization (FISH) staining of the two-colored bacteria unveiled the taxonomic information of the donor endospores that germinated in the recipient’s gut. Using this strategy, we investigated effects of different germinants and pre-treatment interventions on their germination, and found that germination of different commensal bacterial genera was distinctly affected by various types of germinants. This two-color labeling strategy shows its potential as a versatile tool for visually monitoring endospore germination in the hosts and screening for new interventions to improve endospore-based therapeutics.

## Introduction

Many Gram-positive bacteria propagate by forming and spreading endospores. Recalcitrant to desiccation and most disinfectants, the production of endospores permits long-term survival of the microbes in hostile environments.^1,2^ Bacterial sporulation is initiated by an asymmetric cell division through the formation of a polar septum; after this prespore is engulfed by the mother cell, several durable proteinaceous layers are then assembled onto the forespore surface,^3^ which protect them from being lyzed by enzymes. The forespore then matures after its chromosome is saturated with small, acid-soluble proteins and cytoplasm partially dehydrated, enabling endospores’ resistance to UV radiation and heat. The mature endospores can then be released, and ubiquitously found in soil, water, air and almost all human surroundings.^4–6^ People can unintentionally inhale or ingest endospores, and their germination into vegetative bacteria in the respiratory or gastrointestinal tracts can profoundly affect our health.

The spreading and transmission of endospores, on one hand, play an essential role in the pathogenesis of several serious infections caused by bacterial pathogens, such as *Bacillus anthracis* and *Clostridioides difficile*. Endospores of *B. anthracis* can cause cutaneous and systemic anthrax through skin-entry via preexisting lesion or gastrointestinal tracts by ingestion, which is often highly lethal and can be associated with bioterrorism.^6^ As the leading cause of nosocomial diarrhea, *C. difficile*’s ability to sporulate and regerminate within the patients’ intestines leads to the high relapse rate of the infection.^7^ On the other hand, endospores’ transmission can be beneficial to our health. Endospores can be transmitted via fecal-oral route in daily life and easily spread between people living in the same environment, like family or community members.^4,5^ This transmission promotes health through replenishing and maintaining microbial diversity of host’s commensal microbiota,^8^ and preventing the host from pathogens’ infection via conferring colonization resistance,^9^ which is of particular importance for infants.^10^ Moreover, endospores collected from healthy donors’ fecal microbiotas were recently applied in phase III clinical trials to treat *C. difficile* infection via oral delivery, where satisfying outcomes were observed.^11^

**Figure 1.**
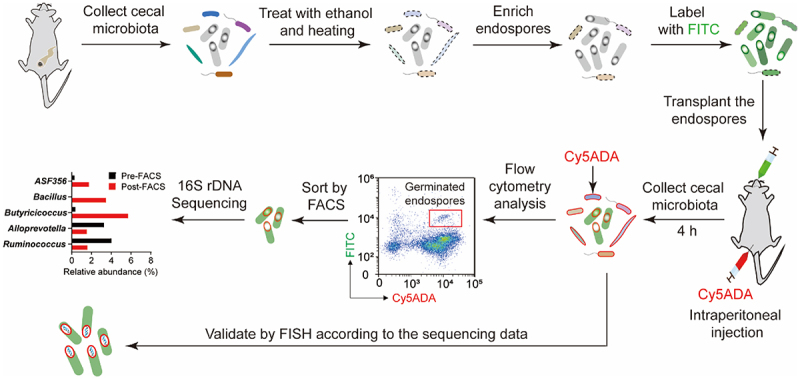
Schematic illustration of the two-step labeling strategy for monitoring endospores’ germination in the mammalian gut. Donor endospores collected from mice cecal microbiotas, which had been treated with 75% ethanol and heating, were labeled with FITC and then given to the recipient mouse by gavage. Cy5ADA probe was simultaneously administered by intraperitoneal injection for gut microbiota labeling. The recipient’s gut microbiota was collected, and analyzed by confocal fluorescence microscopy and flow cytometry. The bacteria co-labeled by FITC and Cy5ADA probes were the germinated endospores, which were then sorted and analyzed by 16S rDNA sequencing. Visual validation of the germinated endospores was conducted by FISH-staining.

The endospores themselves, however, are mostly inactive or nontoxic to host. They must germinate to vegetative form before beneficial or virulence factors can be expressed.^7^ Previous studies have found that foreign *Bacillus clausii* and *C. difficile* endospores could reach the gastrointestinal tract and adhere to intestinal mucosa,^12,13^ but their probiotic function or pathogenicity could not be evaluated properly without recognizing the germination. Therefore, being able to monitor germination *in vivo* is a key step in furthering our understanding of endospore’s *in vivo* activities and functions. Moreover, most of the existing studies focused on the germination of specific pathogens’ endospores in the gut to investigate food safety^14^ and intestinal infections,^15,16^ but commensal microbiota endospores’ transmission and germination still remain poorly understood. In this study, we developed a two-color tagging strategy using fluorescein isothiocyanate (FITC) staining and fluorescent D-amino acid (FDAA)-based metabolic labeling to track the donor and recipient microbiotas (schematic illustration shown in Figure 1), respectively, which allowed visual monitoring and quantitative analysis of the microbiota endospore’s germination in the mammalian gut.

## Results

### Fluorescent staining of bacterial endospores

**Figure 2.**
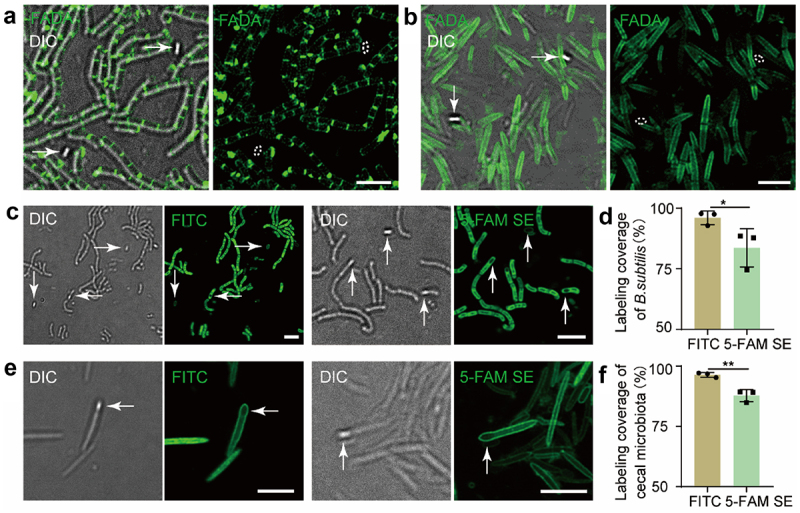
Fluorescence analyses of the bacterial endospores labeled by FADA, FITC and 5-FAM SE. (a) Confocal fluorescence imaging of *B. subtilis* endospores (indicated by arrow) labeled by FADA *in vitro*. DIC, differential interference contrast. Scale bar, 5 μm. (b) Confocal fluorescence imaging of cecal endospores labeled by FADA *in vivo*. Scale bar, 5 μm. Dashed lines in florescence channel indicate the endospores’ positions. (c) Confocal fluorescence imaging of *B. subtilis* endospores (indicated by arrow) stained by FITC (two graphs on the left) and 5-FAM SE (two graphs on the right) *in vitro*. Scale bar, 5 μm. (d) Statistical analysis of the labeling coverage for *B. subtilis* endospores, together with bacteria stained by FITC and 5-FAM SE *in vitro*, Mean ± s.d. are presented for n = 3. (e) Confocal fluorescence imaging of cecal endospores (indicated by arrow) stained by FITC (two graphs on the left) and 5-FAM SE (two graphs on the right) *in vitro*. Scale bar, 5 μm. (f) Statistical analysis of the labeling coverage for cecal endospores, together microbiota stained by FITC and 5-FAM SE *in vitro*, respectively. Mean ± s.d. are presented for n = 3. Representative images of germinated endospores from at least three independent experiments are presented.

To track their germination *in vivo*, donor endospores need to be efficiently labeled. Previously, FDAAs, a type of metabolic labeling probes that can be incorporated into bacterial peptidoglycans (PGNs) via the functioning of D,D- or L,D- transpeptidases,^17,18^ have been applied to fluorescently label various types of bacteria and gut microbiota with high efficiency.^19,20^ Because endospores are also surrounded by a thick layer of PGNs, we hypothesized that endospores could also be labeled by FDAAs. *Bacillus subtilis*, a model species for endospore research, was first tested in labeling with FAM-amino-D-alanine (FADA). After 48 h of labeling, most of the vegetative cells of *B. subtilis* were readily tagged by FADA (0.15 mM), but to our surprise, the endospores could not be labeled (Figure 2(a)). This might be owing to the processing and modifying steps of endospores’ PGNs during the sporulation of *B. subtilis*, which led to different stem peptide structures than their mother cells.^21,22^ We then explored FADA’s labeling efficiency against gut microbiota endospores. FADA (200 µL, 1 mM) was given to the mouse by gavage to metabolically label its gut microbes *in situ*.^20^ Although some sporulating bacteria in the cecal microbiota could be labeled by FADA (**Figure S1**), many of the mature endospores were not labeled (Figure 2(b)). Therefore, FDAA-based metabolic labeling might not be suitable for labeling endospores.

Considering the proteinaceous layers covering bacterial endospores,^23^ we then attempted to label them with fluorescent dyes (FITC and 5-carboxyfluorescein *N*-succinimidyl ester (5-FAM SE)) that were often used in staining proteins. Flow cytometry and confocal fluorescence microscopy analyses indicated that both dyes could stain *B. subtilis* endospores (Figure 2(c)), and FITC showed a higher staining efficiency than 5-FAM SE (Figure 2(d)). Subsequent *in vitro* staining of cecal microbiota endospores was also successful (Figure 2(e)), where the staining efficiency of FITC was higher than that of 5-FAM SE as well (Figure 2(f)). Therefore, FITC was used in the following endospore staining experiments.

**Figure 3.**
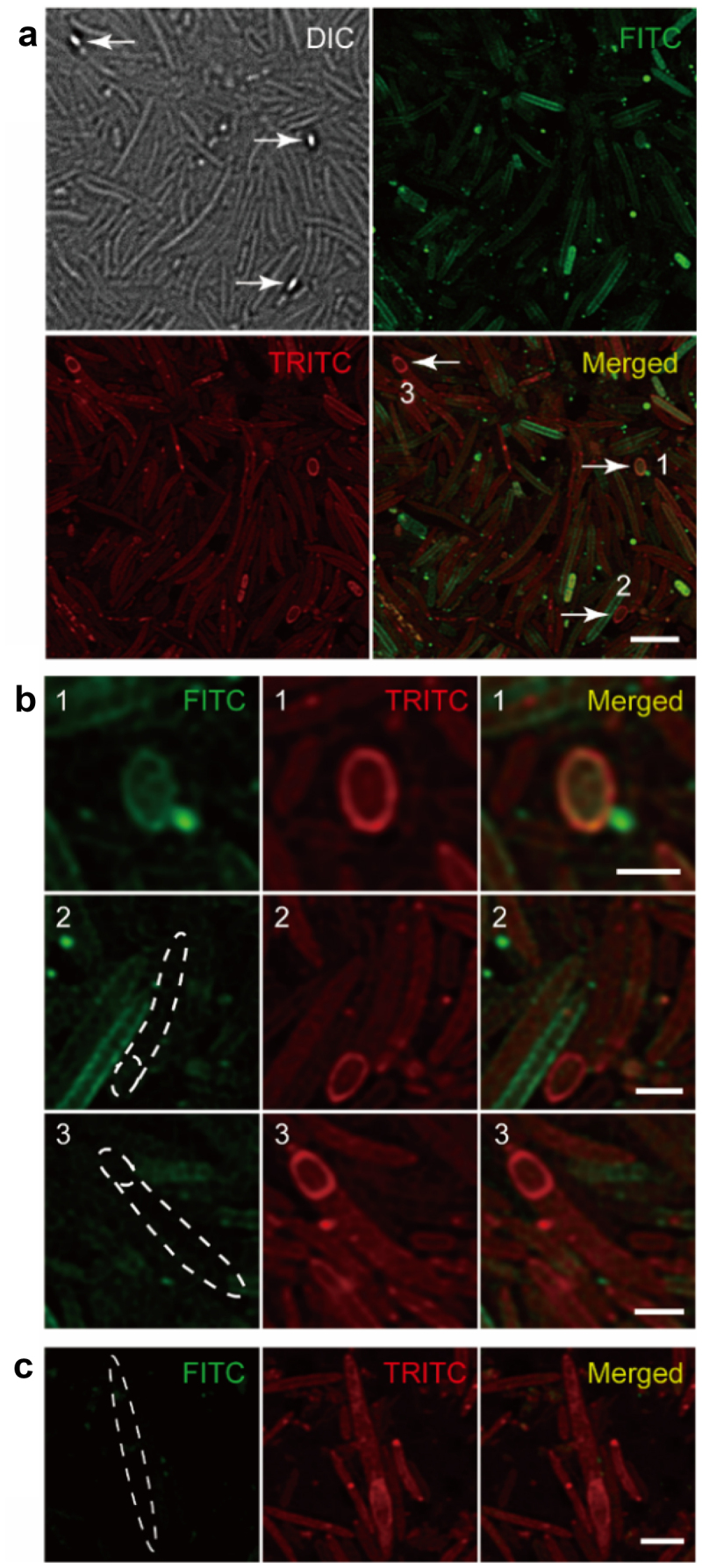
Imaging of bacterial sporulation in the gut recorded via sequential labeling. (a) Confocal fluorescence imaging of the cecal endospores (arrows) sequentially labeled with FITC and TRITC *in vivo*. Scale bar, 5 μm. (b) Zoomed views of the endospores indicated from the merged image above. Scale bars, 2 μm. (c) Two-color fluorescence imaging of a sub-terminal endospore. Scale bar, 2 μm. Representative images of germinated endospores from at least three independent experiments are presented.

We then tested whether FITC could also stain cecal microbiota endospores *in vivo*, the success of which might allow probing the *in vivo* sporulation process. To this end, mice were administered with FITC by gavage; more than 93% of cecal microbes together with the endospores were labeled by FITC *in situ* in 4 h (**Figure S2**). Potentially being unstable in the gastrointestinal environment, isothiocyanate’s high labeling coverage of gut microbes was somewhat surprising. Next, inspired by recent works of our group,^24,25^ where two FDAAs were given to mouse to sequentially label its gut microbiota, we then carried out sequential gavage of FITC and tetramethylrhodamine isothiocyanate (TRITC), in attempt to longitudinally record endospores’ sporulation process. As shown in Figure 3, some mature endospores could be stained by both FITC and TRITC (No. 1), while more endospores were only tagged by TRITC (No. 2, 3). This was probably because the local concentration of TRITC (the second probe applied) was higher than that of FITC, and some of these endospores might be newly synthesized. A recent study showed that many species belonging to the *Firmicutes* phylum could produce endospores with different morphologies *in vitro*.^26^ Here, various patterns of fluorescently labeled endospores could be observed, including mature endospores with mother cells lysed (top row in Figure 3(b)), terminal endospores (middle and bottom rows in Figure 3(b)), and sub-terminal endospores (Figure 3(c)). Taken these data together, the isothiocyanate-derivatized fluorophores could not only be used for endospore labeling, but also for sporulation monitoring.

### Visualizing endospore germination *in* *vitro* via FDAA-labeling

**Figure 4.**
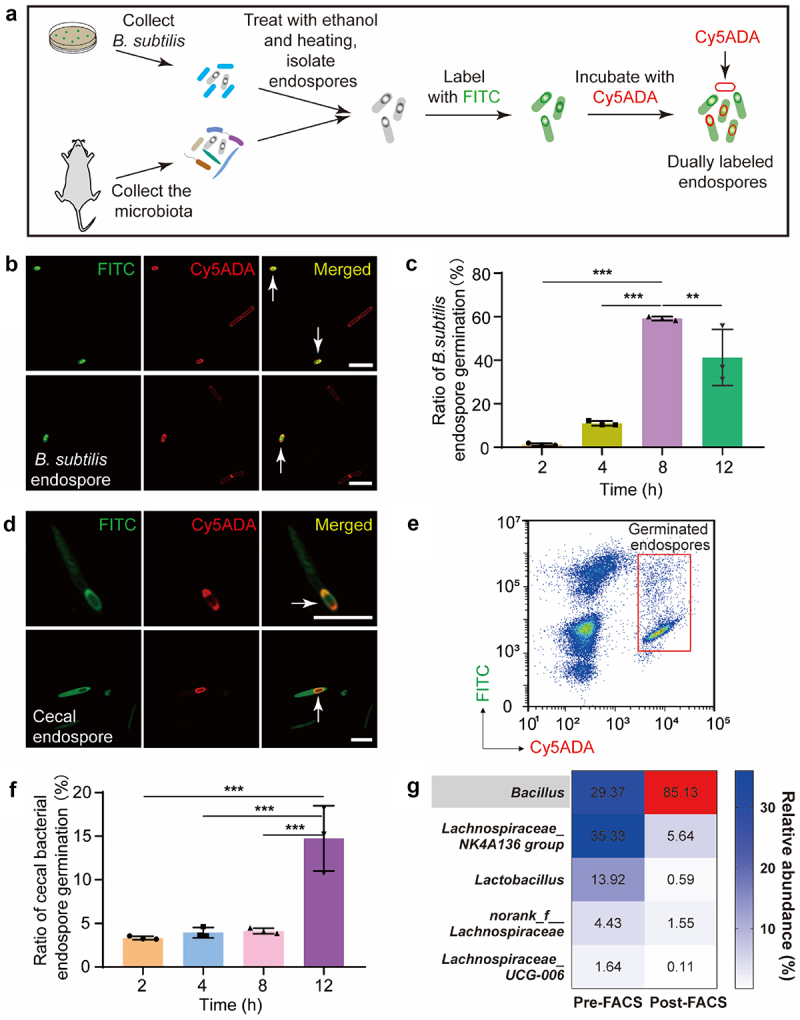
Evaluation of endospores’ germination *in vitro* by two-step tagging with FITC and Cy5ADA probes. (a) Schematic illustration of the assessment of endospores’ germination *in vitro*. (b) Two-color fluorescence imaging of the *B. subtilis* endospore (arrows) germination *in vitro*. Scale bar, 5 μm. (c) Statistical analysis of the labeling coverage for germinated *B. subtilis* endospores. Mean ± s.d. are presented for n = 3. (d) Two-color fluorescence imaging of the cecal endospore (arrows) germination *in vitro*. Scale bar, 5 μm. (e) Flow cytometry analysis of the germinated cecal endospores. The germinated endospores having both FITC and Cy5ADA labeling signals were sorted by FACS. (f) Statistical analysis of the ratios for germinated cecal endospores. Mean ± s.d. are presented for n = 3. (g) 16S rDNA sequencing analysis of the cecal endospores before and after sorting uncovered that *Bacillus* was the major germinated bacteria after 12 h of incubation. Representative images of germinated endospores from at least three independent experiments are presented.

With the endospores fluorescently labeled, we explored whether their germination, which involved the formation of vegetative PGNs, could be probed *in vitro* by FDAA labeling. To avoid the interference of vegetative bacteria, we first ensured that all bacteria but endospores were sterilized. To this aim, a combined treatment of ethanol (75% v/v) and heating (70°C for 20 min) was conducted to spore-forming *B. subtilis*, which was subsequently stained by FITC and then cultured with Cyanine5-tagged D-amino acid (Cy5ADA, 0.15 mM) in Luria-Bertani medium after being washed (scheme shown in Figure 4(a)). Many endospores are known to have strong autofluorescence.^27^ To test whether the autofluorescence could influence the evaluation of endospore germination, we examined the fluorescence spectra of unlabeled endospores with a microplate spectrophotometer (Synergy H1, BioTek). Strong fluorescence was observed when UV light (325 nm) was used for excitation, but very weak fluorescence when visible or near infrared light (488 or 638 nm) was applied (**Figure S3(A,B)**). Therefore, endospores’ autofluorescence would not influence the evaluation of germination using our strategy. Two-color labeled endospores and vegetative cells labeled only with Cy5ADA were observed after 4 h of incubation (Figure 4(b)), indicative of their germination. The ratio of two-colored *B. subtilis* endospores (some lost their refractility under DIC, **Figure S4**), increased significantly in the first 8 h, but declined at 12 h (Figure 4(c), **Figure S5(A)**), which could be because that some of the endospores lost their FITC signals after they germinated. In contrast, no Cy5ADA signals could be observed in incubated endospores which had been further sterilized with NaClO before germination experiment (**Figure S6**).

To monitor the germination of gut microbiota’s endospores *in vitro*, a similar processing of cecal endospores was carried out (scheme shown in Figure 4(a)). Two-color labeled endospores could also be observed (Figure 4(d, e)), and an increasing ratio of germinated endospores was observed during the 12 h incubation (Figure 4(f), **Figure S5(B)**). To investigate the germination capability of different gut bacterial species in the microbiota, we then sorted the germinated endospores (two-colored, shown in Figure 4(e)) by fluorescence-activated cell sorting (FACS). The following 16S rDNA sequencing showed that only the genus *Bacillus* was dramatically enriched in sorted samples (Figure 4(g), **Figure S5(C)**), suggesting that the germinated endospores during *in vitro* incubation were mostly from *Bacillus*.

### Tracking endospores’ germination in the mammalian gut

Bacterial endospores can quickly germinate in a hypertrophic environment and an outgrowth period is often emerged afterward,^28^ causing difficulties in monitoring germination *in vivo*. Taking advantage of the two-color labeling strategy, the efficacy of which was proved via the *in vitro* germination experiment, we then tried to monitor their germination *in vivo*.

**Figure 5.**
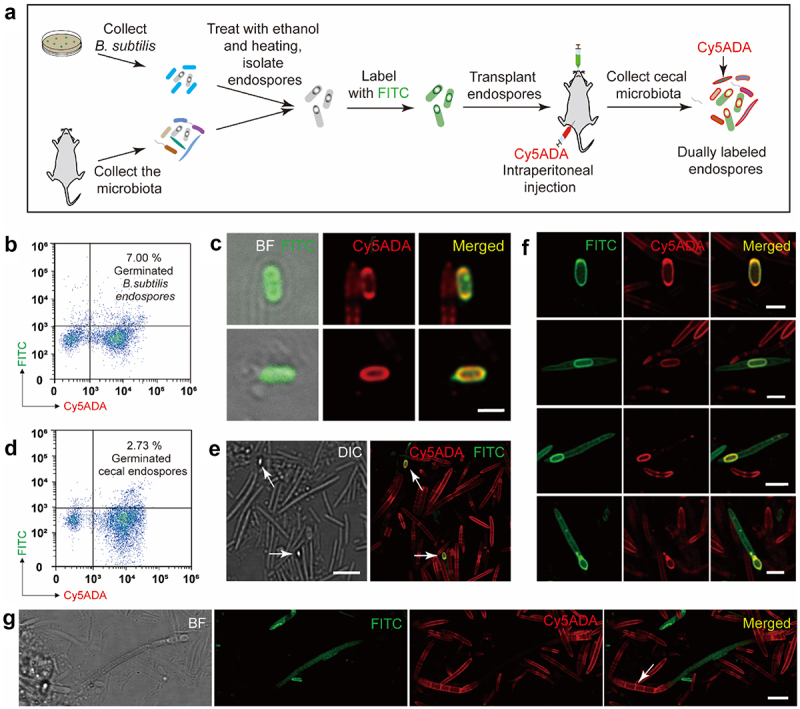
Analyses of endospores’ germination in the gut. (a) Schematic illustration of the procedures for assessing endospores’ germination in the gut. (b) Flow cytometry analysis of the germinated *B. subtilis* endospores from the recipient mice. The upper right sector indicates the *in vivo* germinated endospores, which accounts for 7.0% of the recipient mice’s microbiota. (c) Confocal fluorescence imaging of the *in vivo* germinated *B. subtilis* endospores. Scale bar, 2 μm. (d) Flow cytometry analysis of the germinated cecal endospores from the recipient mice. Cells distributed at the upper right corner indicates the *in vivo* germinated endospores, which accounts for 2.73% of the recipient mice’s microbiota. (e) Confocal fluorescence and DIC imaging of the germinated endospores (arrows) *in vivo*. Scale bar, 5 μm. (f) Representative fluorescence images of *in vivo* germinated endospores having different morphologies. Scale bars, 2 μm. (g) Confocal fluorescence imaging of germinated SFB endospores (arrow) in the gut. Scale bar, 2 μm. Representative images of germinated endospores from at least three independent experiments are presented.

First, FITC stained *B. subtilis* endospores were given to the recipient mice by gavage (200 µL, ~5×10^8^ endospores), and Cy5ADA probes (150 µL, 1 mM) was simultaneously administrated via intraperitoneal injection (scheme shown in Figure 5(a)). The injected Cy5ADA could be discharged into the intestines through bile acid secretion, leading to the quick and lasting labeling of gut microbiota *in situ*.^29^ Flow cytometry analysis of the cecal microbiota (collected 4 h after gavage) showed that the dually labeled *B. subtilis* endospores accounted for ~7.0% of the total gut microbes (Figure 5(b)). Fluorescence microscopy analysis also confirmed their germination (Figure 5(c)), in which the FITC signal indicated that they were from the donor endospores, and the Cy5ADA signal implied their survival and germination in recipient’s gut. PGN sites with more active constructions often have stronger labeling of FDAA,^30^ and therefore, the different distributions and intensities of Cy5ADA among various germinated endospores could reveal their germination processes. Some of the germinated *B. subtilis* endospores showed higher FDAA intensities at the ends (Figure 5(c) first row), while others showed uniform distribution around the cell (Figure 5(c) second row), implying that *B. subtilis* endospores’ PGNs synthesis might start from the ends during germination.

With the successful monitoring of model species’ endospore germination in the gut, we then went on to probe cecal endospores’ germination *in vivo*. Cecal endospores labeled by FITC *in vitro* were transplanted to the recipient mice (200 µL, ~5 × 10^8^ endospores), which also received Cy5ADA probes (150 µL, 1 mM) simultaneously. Their cecal microbiotas were then collected in 4 h (scheme shown in Figure 5(a)), and the dually labeled endospores could be detected by both flow cytometry (Figure 5(d)) and fluorescence microscopy (Figure 5(e)). The germinated endospores were morphologically diverse, including mature endospore without mother cells (first row in Figure 5(f)), central (second row), sub-terminal (third row), and terminal endospores (fourth row). Most of the germinated endospores were uniformly labeled with FDAA throughout the cell wall (second and third rows in Figure 5(f), **Figure S7(A)**), while some showed strong FDAA-signals at one end (**Figure S7(B)**) or both ends (first row in Figure 5(f) and **Figure S7(C)**). To the best of our knowledge, this is the first report that visually recorded the endospore germination processes *in vivo*.

Of special note, as a heavily studied spore-forming bacterial species, segmented filamentous bacteria (SFB) play important roles in inducing T helper cell 17, and many other immunity-related effects.^31,32^ In the mammalian gut, SFB normally attach to the ileal epithelium, extend from its distal end and release endospores from the maturing filaments,^33^ which facilitates its vertical transmission from parents to offspring and horizontal transmission between hosts.^34,35^ Two intracellular offsprings formed within a differentiating filament can be released into the intestines and function as endospores.^34^ Intriguingly, here we observed an intersegmental SFB germinating in the gut (Figure 5(g)), the labeling pattern of which was very different from these observed in other gut bacteria (Figure 5(f)). The Cy5ADA-labeled segments were probably owing to several pairs of intracellular offsprings that germinated within their mother cells. It was also possible that these cells already underwent one cell division, considering their uniform labeling signals on the cell walls and the fact that no traces of their mother cells could be observed. The life-cycle of SFB has been investigated mostly by scanning electron microscopy analysis of the mammalian intestine samples.^33^ Our labeling strategy provides a unique perspective to study their activities and transmission in the gut.

### Evaluation of germinants’ effects on cecal endospore’s germination in the gut

**Figure 6.**
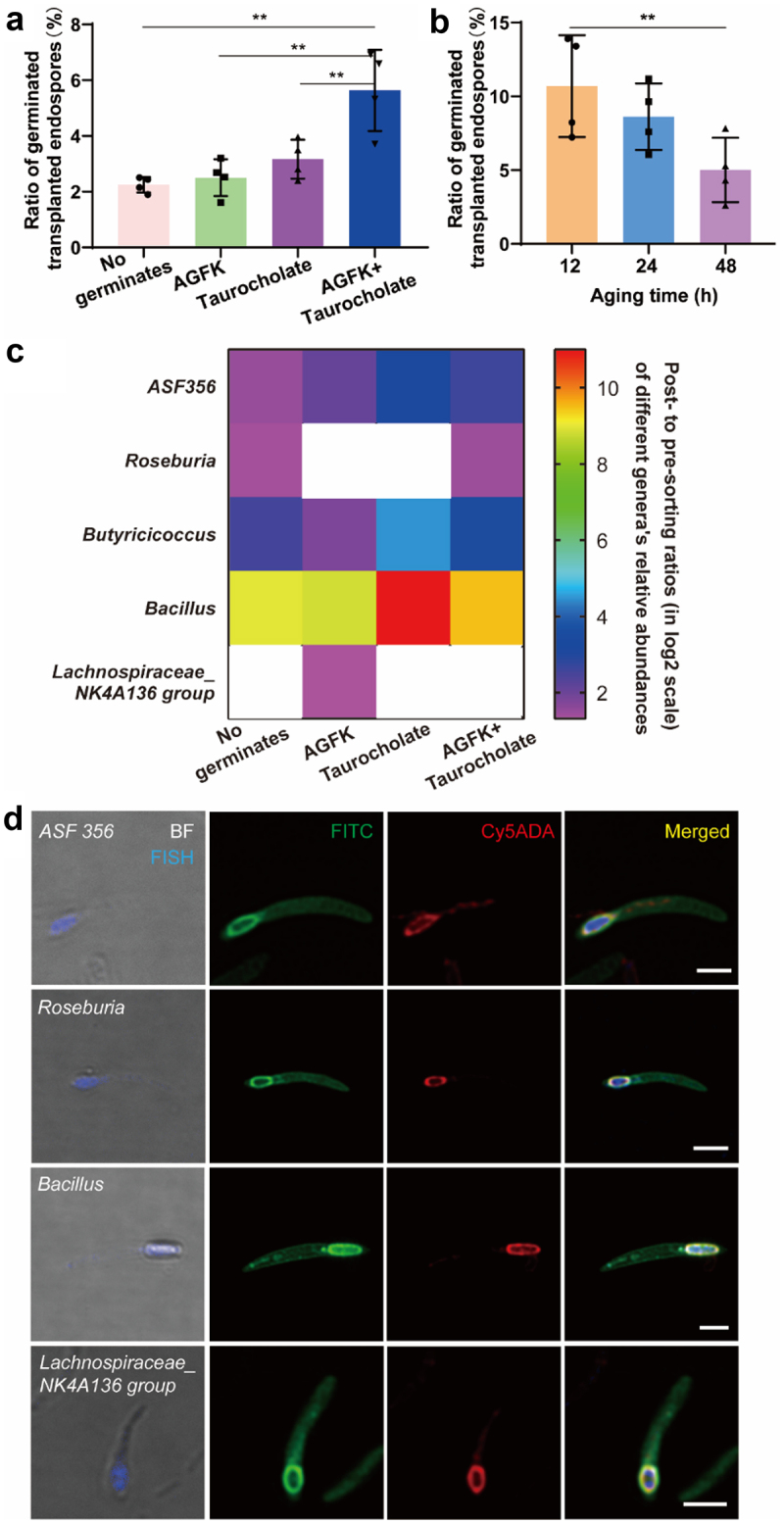
Evaluation of the effects of germinants and aging-time on cecal endospores’ germination in the gut. (a) Statistical analysis of the ratios of germinated cecal endospores in the gut supplied with different germinants. Mean ± s.d. are presented for n = 4. (b) Statistical analysis of the ratios of germinated cecal endospores in the gut after aging for 12, 24 and 48 h. Mean ± s.d. are presented for n = 4. (c) Heat map showing the enrichment of different bacteria after cell sorting. The post- to pre-sorting ratios (in log_2_ scale) of different genera’s relative abundances were shown; blank spots mean no enrichments. (d) Confocal fluorescence imaging of the dually labeled and FISH-stained spores belonging to four genera. The germinated cecal spores in mice received two-step labeling of FITC and Cy5ADA were stained by different FISH probes (blue) targeting corresponding genera. Scale bars, 2 µm. Representative images of germinated endospores from at least three independent experiments are presented.

Endospore germination is triggered by nutrients, and can be promoted by amino acids, sugars, purine nucleosides^28,36^ and primary bile salts,^37^ which are often defined as germinants. The improvement of endospore germination can be beneficial for probiotics endospore colonization^11^ and pathogenic endospore elimination.^38^ Here we evaluated the influences of two germinants AGFK (composed of L-asparagine, D-glucose, D-fructose, KCl and L-alanine) and taurocholate on cecal endospore’s germination in the gut. Compared with the control group (no germinants added), the germination was slightly improved when AGFK and taurocholate were supplied separately; when they were applied together, the improvement was significant (Figure 6(a), **Figure S8(A)**), indicative of a synergistic effect when different germinants were combined.

Previous study has found that *in vitro* germination of *B. subtilis* can be affected by endospores’ age,^39^ but few research has examined the effects of microbiota endospores’ age on their germination *in vivo*. For this purpose, FITC stained cecal endospores were transplanted to the different groups of recipient mice 12, 24 and 48 h after they were isolated, respectively, and FDAA labeling was then carried out as described above. The stability of FITC labeled endospores were first examined before performing transplantation, and no apparent changes of labeling ratio, fluorescent intensity (**Figure S9**), or spontaneous germination (**Figure S10**) were observed during the 48 h of storage. Flow cytometry analysis indicated that the ratios of germinated endospores decreased as the donor aged in the 48 h period (Figure 6(b)), suggesting that cecal endospores might lose some of their germination capability as they age. Therefore, the shelf life of prebiotic endospores and how to maintain their germination abilities merit further study.

A recent study has shown that many bacterial species of the *Firmicutes* (the major Gram-positive bacteria) in the human gut microbiota could produce endospores when cultured *in vitro*.^26^ To determine which bacterial groups could germinate in the mammalian gut, we sorted (by FACS) the dually labeled endospores from the microbiotas of mice treated with different germinants. The following 16S rDNA sequencing of the sorted bacteria showed that, compared with the pre-sorting samples, several bacterial genera were enriched, including *ASF*356, *Roseburia, Butyricicoccus, Bacillus* and *Lachnospiraceae_NK4A136* group (Figure 6(c), **Figure S8(B)**), indicative of their successful germination in the gut. In sharp contrast, we only detected the germination of *Bacillus* when cecal endospores were incubated *in vitro* (Figure 4(g)), suggesting that the intestinal environment was essential for the successful germination of many cecal endospores. Regarding different germinants, compared with the group receiving no germinants, AGFK promoted *Lachnospiraceae*_*NK4A136*, and taurocholate improved *ASF*356, *Butyricicoccus* and *Bacillus* germination, respectively (Figure 6(c)). When two germinants were used together, *ASF*356 and *Bacillus* germination were also improved but no apparent influences were observed on *Butyricicoccus* and *Lachnospiraceae_NK4A136*. Therefore, germinants’ effects on different bacterial groups’ germination in the gut varied.

With the taxonomic compositions of germinated cecal endospores determined, we then visually identified the germination process of different bacteria. To this end, we resorted to fluorescence *in situ* hybridization (FISH) to stain the germinated endospores of different genera. Four FISH probes (Table S1) were applied based on the sequencing data, including two probes newly designed in this study (staining specificities verification data shown in **Figure S11**). Because many of the germinated endospores were still in the mother cells (Figure 5(f)), we treated the samples with lysozyme and mutanolysin to enhance the cell permeability. Representative images of the labeled endospores for each genus were presented in Figure 6(d). Most of the germinated *ASF*356 (**Figure S12**), *Roseburia* (**Figure S13**), *Bacillus* (**Figure S14**) and *Lachnospiraceae_NK4A136* (**Figure S15**) cells were terminal endospores. The germination of *Roseburia* and *Bacillus* aligns with previous studies, where resilient *Roseburia* collected from feces and model species of *Bacillus* could germinate *in vitro*,^26,36^ but germination of *ASF*356 and *Lachnospiraceae_NK4A136* endospores have never been reported. Thus, this integrative strategy using *in vivo* labeling, cell sorting, DNA sequencing and FISH staining enables studying the germination of various endospores that may not be investigable *in vitro.*

## Discussion

Like many other microbial processes that occur constantly but unnoticed in our gut, germination of endospores *in vivo* has not been a subject that can be directly monitored. During the development of a milestone endospore therapeutic (SER-109, composed of *Firmicutes* endospores from healthy volunteers for treating *C. difficile* infection), it was debated whether a dose-dependent effect existed, and this disagreement resulted in a failed phase II trial^40,41^ and finally a successful phase III trial partially because of a 10-fold dose increase.^11^ This endorsed the necessity and importance of furthering our understanding of the endospores’ germination processes *in vivo*. Here, we developed a two-step labeling strategy for visualizing the *in vivo* germination of endospore from specific bacteria or commensal microbiota, and assessing the effects of different germinants or preconditioning treatments on endospore germination. How endospore germinates *in vivo* is an important topic in infection pathobiology, and this new strategy can greatly deepen the pathobiology studies via directly looking at which and how endospores germinate *in vivo*. The fact that endospores from many commensal bacteria, which function to maintain gut homeostasis,^42,43^ can successfully germinate in the mammalian gut, suggests that transmission and germination of endospores may indeed exert beneficent influence on host health. Intriguingly, the germination of SFB, an immunologically important commensal bacteria, was accidentally observed by our dually-labeled strategy, which directly confirmed the inter-host transmission of SFB via endospores. This finding offers new perspectives on furthering our understanding of the microbiology of this special bacteria, and will be useful for manipulating host immunity via efficiently transplanting and colonizing SFB. In addition, the observed varied germination modes of different cecal endospores may broaden our knowledge of this basic microbial process. This strategy also provides an opportunity to quantify the endospores’ germination *in vivo*. The use of this two-step tagging strategy in evaluating the effect of germinants on endospore germination, can facilitate the screening of germinants/preconditioning techniques to regulate endospore germination. This may help the development of more effective strategies for preventing pathogenic endospores’ transmission between humans, and treating infections caused by spore-forming bacteria, according to the “germination to eradicate” strategy.^42^ Our work provides a versatile and powerful method for monitoring endospore germination *in vivo*, and potentially facilitates endospore’s utility in improving human health.

In summary, via a two-color labeling strategy, where the donor endospores and their subsequent germination in the mammalian gut were fluorescently labeled by two different methods, we successfully imaged, for the first time, the germination of different bacterial genera contained in gut microbiota in their new hosts’ intestines, and assessed the effects of different germinants and pre-treatment interventions on their germination. This new technique will be of great value in furthering our understanding of this ubiquitous microbial process that are tightly related to human health.

## Materials and methods

### Reagents

The FITC and TRITC fluorescent dyes were purchased from Aladdin Biochemical Technology (Shanghai, China), 5-FAM SE was purchased from MeloPEG Company (Shanghai, China). FDAA probes were synthesized by Chinese Peptide Company (Hangzhou, China). FISH probes were bought from Sangon Biotech (Shanghai, China). All the culture mediums were purchased from Qingdao Hope Biotechnology (Qingdao, China), and Schaeffer’s liquid medium was prepared according to a previous report.^44^

### Bacteria strain and mice

*B. subtilis* (CICC 23659) was obtained from China Center of Industrial Culture Collection (Beijing, China). Six-week-old C57BL/6 specific pathogen free (SPF) male mice were purchased from Jie Si Jie Laboratory Animals (Shanghai, China) and fed in a temperature-controlled (25°C) facility in the animal facility of Renji Hospital. Each group of mice was fed in a separated cage and supplied with a standard chow diet and clean water. All animal experiments were performed under guidelines approved by the Institutional Animal Care and Use Committee of the Shanghai Jiao Tong University School of Medicine.

### *B.* *subtilis* endospore preparation and cecal microbiota’s endospore collection

*B. subtilis* was cultured in Schaeffer’s liquid medium at 37°C for 96 h in an incubated shaker, then centrifuged at 4000 × g for 5 min and washed three times with phosphate buffer saline (PBS). Then a sequential processing of 75% ethanol (1 h) and heating (70°C, 20 min) was applied to kill the vegetative bacteria. Density gradient centrifugation was applied to enrich the endospores. Two mL of *B. subtilis* suspension, and equal-volumes of 35% and 65% sucrose solutions were pipetted into the bottom of a tube sequentially, which were then centrifuged at 1400 × g for 5 min. The second and fourth layers of the precipitates were pipetted to collect the isolated endospores.

SPF C57BL/6 mice were sacrificed by cervical dislocation to collect their gut microbiotas. Briefly, the cecum was dissected with a pair of iris scissors in 2 mL sterile PBS, the mixture of minced tissues and digesta was then filtered with a 40 μm cell strainer to remove most of the debris. The filtered mixture was centrifuged at 4000 × g (5 min) to obtain pellet and washed twice with PBS. Next, sterilized and isolated the cecal endospores by density gradient centrifugation as precedingly described.

### *In vitro* labeling of *B.* *subtilis* endospores with FADA

*B. subtilis* was labeled with 0.15 mM FADA probes in the dark during 48 h of incubation in Schaeffer’s liquid medium. Labeled *B. subtilis* bacteria and endospores were collected after 4 h and 48 h incubation respectively, and then analyzed with confocal fluorescence and DIC microscopy (Leica TCS SP8, Solms, German).

### *In vitro* staining of endospores with FITC and 5-FAM SE

The enriched *B. subtilis* and cecal endospores were suspended in 1 mL of 0.1 M sodium bicarbonate (OD_600_ = 1.5, pH = 9.0), and stained with 2 mM of FITC or 5-FAM SE, respectively, for 1 h in the dark under room temperature. The stained endospores were then centrifuged at 4000 × g for 5 min, washed three times with sterile PBS and stored at 4°C for the following *in vitro* germination or oral transplantation experiment. Flow cytometry and confocal fluorescence microscopy analysis were used to evaluate the staining efficiency.

### *In vivo* labeling of cecal microbiota’s endospores with FADA, FITC and 5-FAM SE

The SPF C57BL/6 mice received 200 µL 1 mM FADA, 2 mM FITC or 5-FAM SE by gavage, respectively. The mice were then sacrificed by cervical dislocation 4 h later, and their labeled cecal microbiotas were collected and analyzed according to the procedures mentioned above. A sequential staining of FITC and TRITC was conducted to probe the bacteria sporulation in the gut. SPF C57BL/6 mice received 200 μL of 2 mM FITC in PBS by gavage, and a second 200 μL of 2 mM TRITC was performed with an interval of 4 h. Two hours later, cecal microbiotas of the mice were collected, washed and fixed according to the preceding steps.

### Evaluation of endospores’ auto-fluorescence

The isolated and FITC labeled cecal endospores were separately resuspended in PBS (OD_600_ = 0.5), and the fluorescence spectra of these endospores were examined via a microplate spectrophotometer (Synergy H1, BioTek). Endospores’ fluorescence spectra was collected with 2 excitation wavelengths at 300 and 488 nm respectively, and the emission spectra measurements were collected between 325 and 700 nm (20 nm steps). Five spectra were collected for each sample and the resultant spectra were summed to form the final fluorescence spectra.

### *In vitro* labeling of endospore germination

FITC stained *B. subtilis* endospores were incubated in Luria-Bertani broth (OD_600_ = 0.5) with 0.15 mM Cy5ADA probes in an incubator; FITC-stained cecal endospores were incubated in modified Gifu anaerobic liquid medium (OD_600_ = 0.5, GAM broth supplied with 0.06 mg/mL L-tryptophan, 0.3 mg/mL L-arginine, 0.003 mg/mL hemoglobin and 0.00015% vitamin K1) with 0.3 mM Cy5ADA probes in an anaerobic chamber (Concept 400, Baker Ruskinn, UK) filled with an atmosphere of 80% nitrogen, 10% carbon dioxide and 10% hydrogen.^45^ The incubated *B. subtilis* and cecal endospores were collected every 2 h and fixed with 2% paraformaldehyde after being washed twice with sterile PBS. Then the endospore germination was evaluated by flow cytometry and confocal fluorescence microscopy.

In order to test whether Cy5ADA probe could label non-germinated endospores, the *B. subtilis* endospores were treated with 0.5% NaClO for 10 min at room temperature, these inactive *B. subtilis* endospores were then incubated with Cy5ADA in Luria-Bertani broth at 37°C for 8 h. The Cy5ADA labeling of inactive endospore was evaluated by flow cytometry.

### *In vivo* labeling of endospore germination

FITC stained *B. subtilis* or cecal endospores from donor mice (~5 × 10^8^ spores of each in 200 μL PBS) were administrated to the recipient mice by gavage, and Cy5ADA probe (150 μL, 1 mM) were simultaneously given to the mice by intraperitoneal injection. The cecal microbiotas were collected 4 h later and fixed with 2% paraformaldehyde for 1.5 h at room temperature, then resuspended in 50% ethanol-PBS (v/v) after being washed twice, and stored at −30°C for the following experiments.

### Germinants and aging treatments

Two kinds of germinants were selected to improve *in vivo* endospore germination as follows: AGFK (2.5 mM L-asparagine, 5 mg/mL D-glucose, 5 mg/mL D-fructose, 50 mM KCl and 0.01 M alanine)^46^ and 0.1% taurocholate.^47^ SPF C57BL/6 mice were randomly assigned into four groups, and each group of mice (n = 4) received equal numbers of FITC stained cecal endospores in accompanying sterile PBS, AGFK, 0.1% taurocholate or AGFK + 0.1% taurocholate, respectively. The following steps were the same as preceding two-step tagging procedures. Next, we assessed the effect of endospore aging time on *in vivo* germination activity, cecal endospores were collected and stored at 4°C for 12, 24 and 48 h, respectively, after FITC staining. Then the aged endospores were separately transplanted to different groups of recipient mice. The labeling ratio and fluorescent intensity of these labeled endospores were tested by flow cytometry during the storage to confirm their stability.

To test the possibility of spontaneous germination of gut bacterial endospores during the 48 h storage at 4°C, we added 0.3 mM Cy5ADA probe into the stored endospores, and examined their germination by flow cytometry during the storage.

### Flow cytometry

Flow cytometry analysis and sorting of the dually labeled cecal endospores were performed on CytoFLex (Beckman Coulter Life Sciences, Indianapolis, IN, USA) and Melody cell sorter (BD Biosciences, San Jose, CA, USA), respectively. FlowJo software (V 10.0.8r1) was applied to analyze the data. The labeled cells were identified with flow cytometry plots of logFSC versus logSSC and gated on fluorescence. In total, 15,000 events of each sample were collected for analyses. As to cell sorting, a total of at least 1.5 × 10^6^ two-colored endospores were sorted for each sample for 16S rDNA sequencing.

### Confocal fluorescence microscopy

The labeled bacteria were inoculated onto agarose pads (1.5% w/v in PBS, ~1 mm in thickness) and covered with glass coverslips. A laser scanning confocal microscope (Leica TCS SP8, Solms, German) was applied for fluorescence imaging. Samples were excited with 488 nm for FADA probe, FITC and 5-FAM SE, and 638 nm for Cy5ADA probe. The emission was detected using corresponding emission filters.

### DNA extraction and 16S rDNA sequencing

DNA was extracted using Omega Bacterial DNA Kit (Omega Bio-Tek, Norcross, GA, USA) from cecal bacteria samples before sorting, and from the dually labeled endospores sorted by FACS, respectively. The V3–V4 region of the bacteria 16S rDNA was amplified by PCR according to previous protocols.^25^ Then the amplicons were extracted from 2% agarose gels, purified with an AxyPrep DNA Gel Extraction Kit (Axygen Biosciences, Union City, CA, USA) and quantitated using QuantiFluo-ST (Promega, Madison, WI, USA). The subsequent sequencing was completed with Illumina MiSeq platform (Illumina, San Diego, CA, USA) following the standard protocol.

Raw fastq files were de-multiplexed and quality-filtered using QIIME (version 1.9.1) and operational taxonomic units (OTUs) were clustered with 97% similarity cutoff (UPARSE, version 7.0.1090), chimeric sequences were identified and removed using UCHIME. The taxonomy of each 16S rRNA gene sequence was analyzed by RDP Classifier (http://rdp.cme.msu.edu/) against the SILVA (SSU138) 16S rDNA database with a confidence threshold of 80%.

### Fluorescence *in* *situ* hybridization

The bacterial samples were washed twice with PBS after being stored at −30°C for >24 h, and pre-treated with lysozyme and mutanolysin to improve the permeability of endospores according to previous study.^48^ Then cecal bacteria were suspended in hybridization buffer (0.9 M NaCl, 20 mM Tris (pH 7.5), 0.01% SDS, and formamide, if required) with a final concentration of 5 ng/µL FISH probes, and incubated overnight at required temperature (Table S1) in a ThermoMixer (Eppendorf, Germany). After hybridization, a two continuous washing process (2 × 15 min) at the required washing temperature (**Table S1**) was performed in washing buffer (0.9 M NaCl, 20 mM Tris (pH 7.5), 0.01% SDS). Endospores were then suspended in PBS before analysis with fluorescence microscopy. FISH probe sequences that have been previously reported^49,50^ are listed in Table S1.

Labeling specificities of the two newly designed FISH probes (ASF356-235 and LNK4A) were separately tested against a fixed soil microbiota sample that did not share any genera with the mouse gut microbiota. Probes EUB338 and NONEUB^51,52^ were used as positive and negative controls, respectively. Moreover, flow cytometry was used to analyze the labeling ratios of two genera in the microbiota according to a previous protocol.^47^ The labeling ratios of the probes were compared with their relative abundances obtained by 16S rDNA sequencing, respectively, the agreement of which could indicate high labeling specificities.

## Supplementary Material

Supplemental Material

## Data Availability

The 16S rDNA sequencing data of the cecal microbiotas have been deposited in the Sequence Read Archive with BioSample accessions (PRJNA814837) respectively. Additional data related to this paper may be requested from the corresponding authors.
