# Supplementary material for: Visualizing germination of microbiota endospores in the mammalian gut
Source: Gut Microbes. 2022 Sep 29;14(1):2125737. doi: 10.1080/19490976.2022.2125737 (PMC9543051; doi:10.1080/19490976.2022.2125737)
Supplement: Supplemental Material [file KGMI_A_2125737_SM9667.pdf]

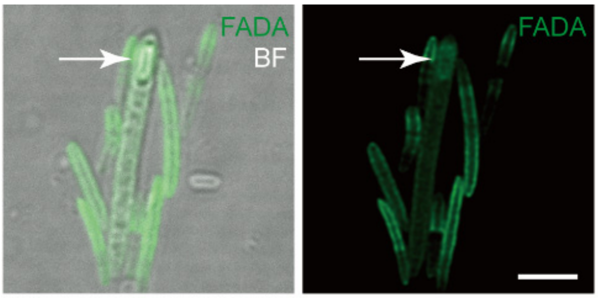

Figure S1. Confocal fluorescence microscopy of the cecal endospore (arrow) labeled by FADA probes *in vivo*. Scale bar, 5 μm. Representative images from at least three independent experiments are presented.

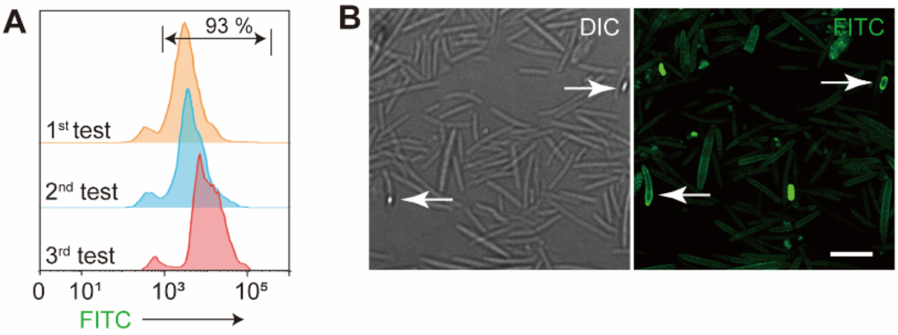

Figure S2. Evaluation of labeling efficiency of cecal microbiota, together with endospores by FITC *in vivo*. (**A**) Flow cytometry analysis of the cecal microbiota labeled by FITC *in vivo*. (**B**) Confocal fluorescence microscopy analysis of the cecal endospores labeled by FITC *in vivo*. Representative images from at least three independent experiments are presented. Scale bar, 5 μm.

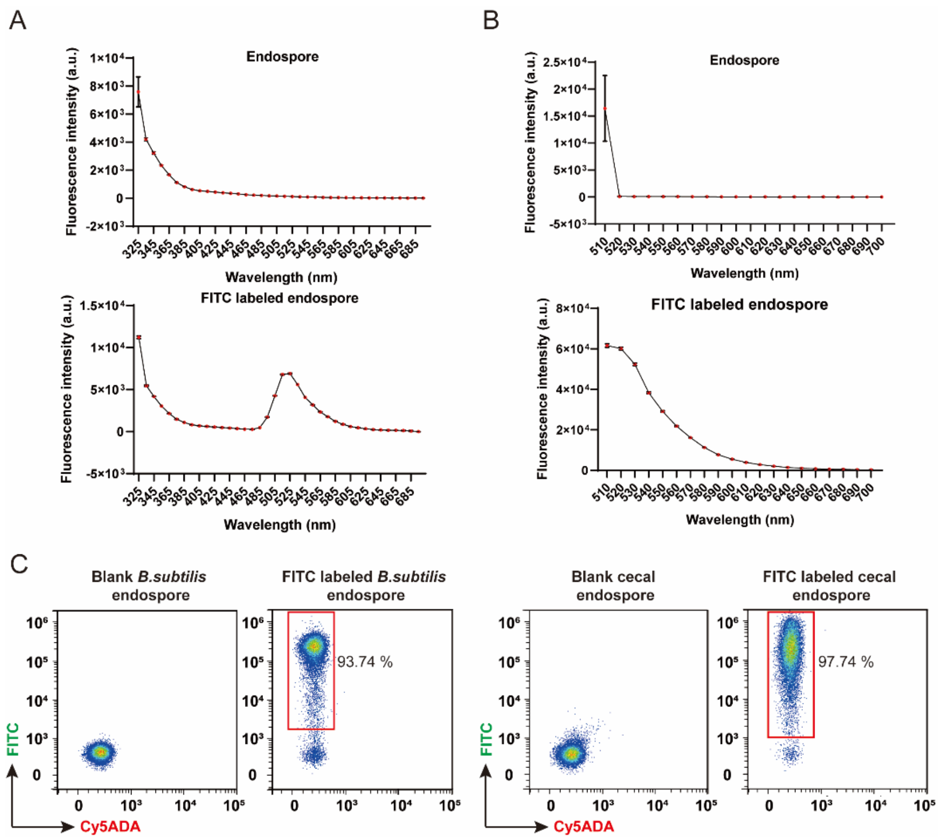

Figure S3. (**A-B**) Analysis of cecal endospore's auto-fluorescence when being excited at 300 nm (**A**) or 480 nm (**B**) via a plate reader. (**C**) Flow cytometry analysis of *B. subtilis* (two charts on the left) and cecal (two charts on the right) endospores labeled without or with FITC.

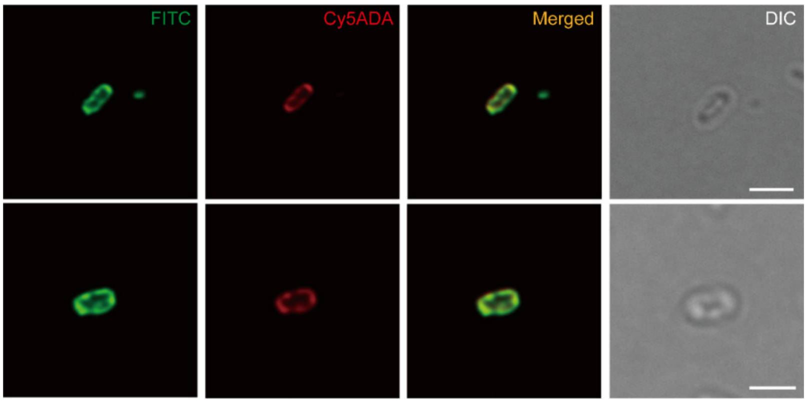

Figure S4. Confocal fluorescence imaging of germinated *B. subtilis* endospores with no apparent refractility. Representative images of dually labeled germinated endospores from at least three independent experiments are presented. Scale bar, 5 μm.

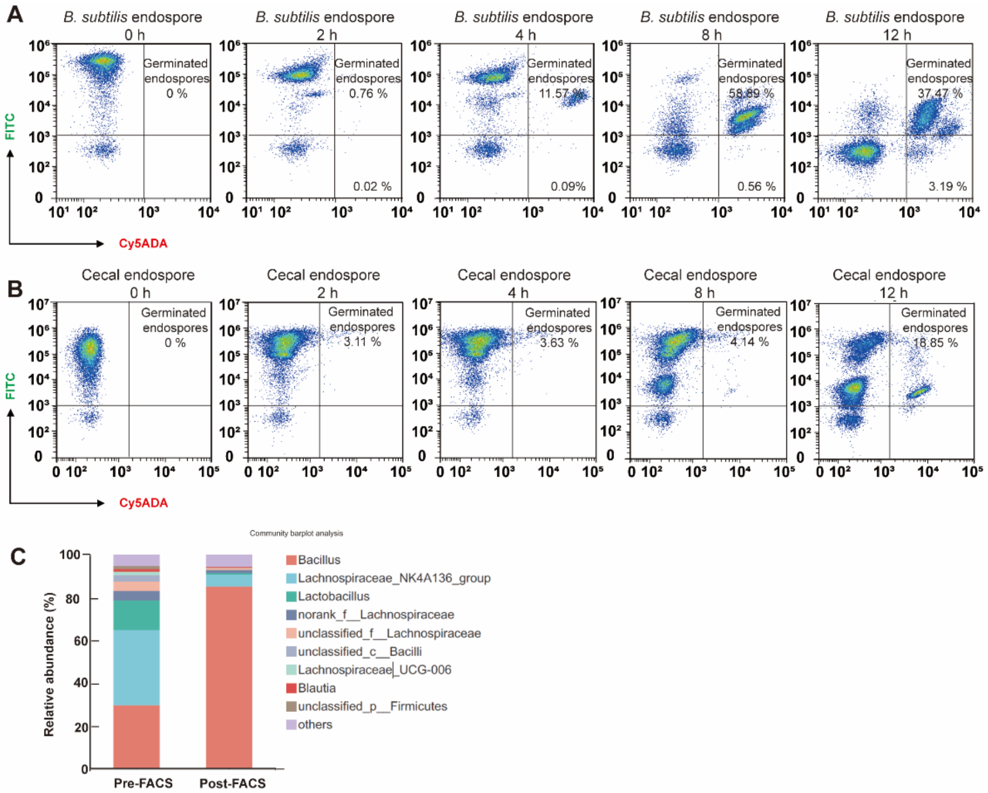

Figure S5. Evaluation of the germinations of *B. subtilis* and cecal endospores *in vitro*. (**A**) Flow cytometry analysis of the *B. subtilis* endospores' germination during 12 h incubation in Luria-Bertani broth. (**B**) Flow cytometry analysis of the cecal endospores' germination during 12 h incubation in modified GAM broth. Representative data from three independent experiments are show. (**C**) 16S rDNA sequencing data of the *in vitro* cultured cecal endospore samples before and after FACS.

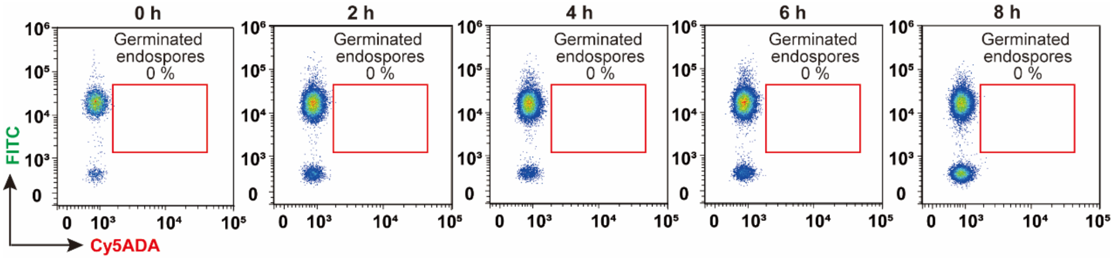

Figure S6. Flow cytometry-based monitoring of the germination of FITC-stained *B. subtilis* endospores (after NaClO treatment), which were labeled with Cy5ADA.

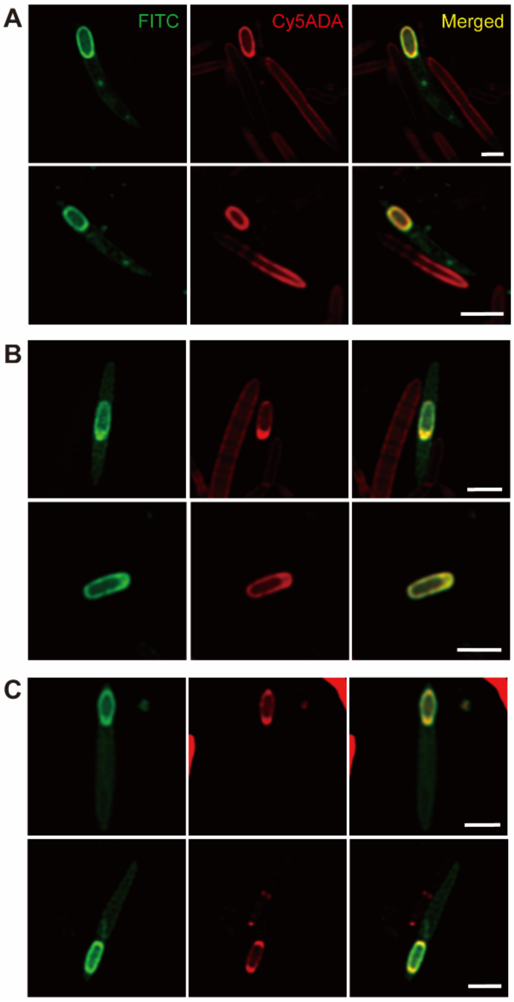

Figure S7. Two-color fluorescence imaging of endospores having different germination patterns. (**A**) Endospores germinate homogeneously throughout the germ cell wall. (**B**) Endospores germinate from one end. (**C**) Endospores germinate from both ends. Scale bars, 2 μm. Representative images from at least three independent experiments are presented.

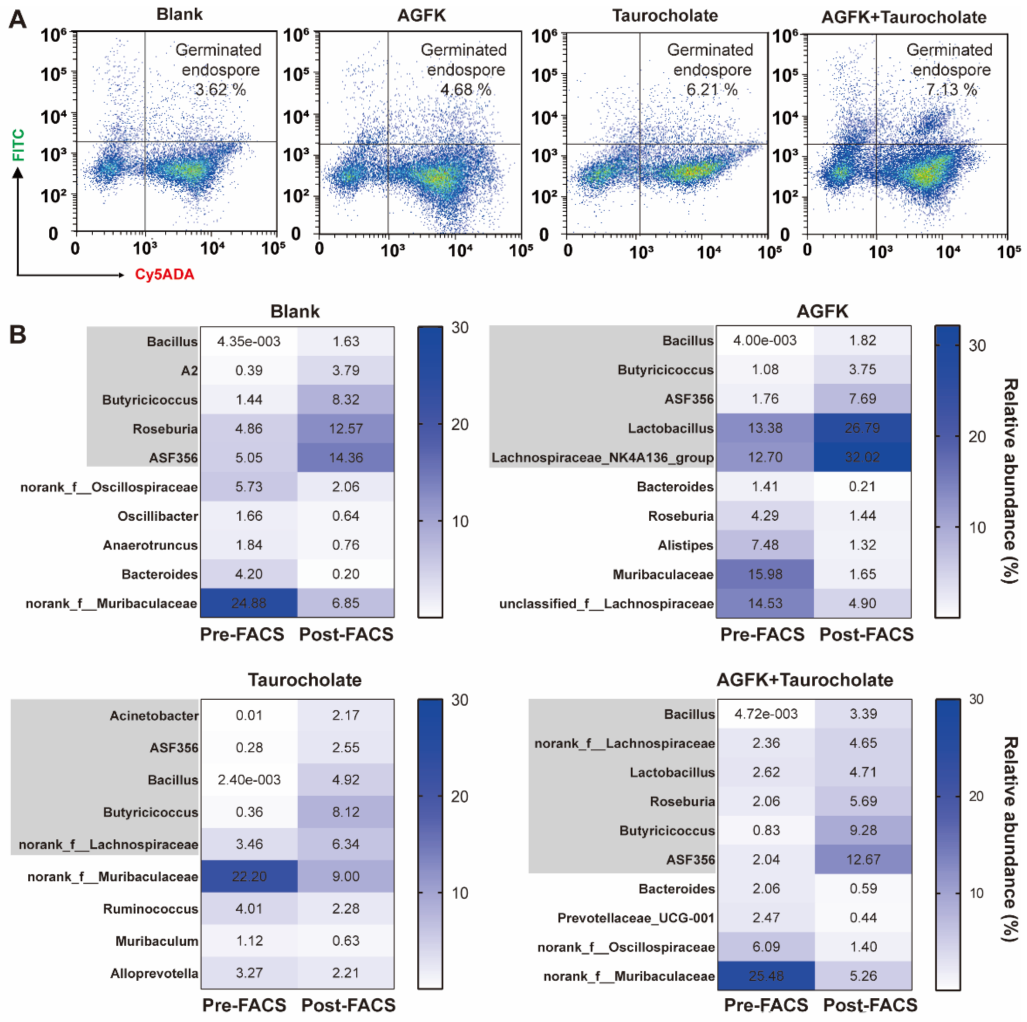

Fiure S8. Flow cytometry and 16S rDNA sequencing analysis of cecal endospores germinated *in vivo*. (**A**) Labeling coverage of endospores germinated *in vivo*, which had both FITC and Cy5ADA labeling signals. (**B**)16S rDNA sequencing analysis of the cecal microbiota before and after FACS revealed that several bacterial genera were enriched in the germinated bacteria. Each square represents a specific taxon, and the number represents the relative abundance of the corresponding genera.

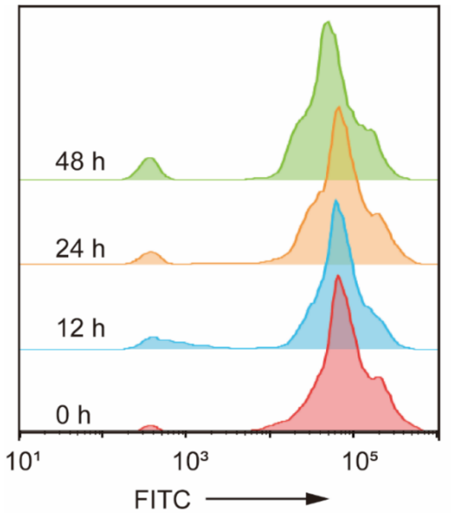

Figure S9. Flow cytometry-based evaluation of the stability of FITC-labeled endospores during 48 h storage at 4°C in PBS.

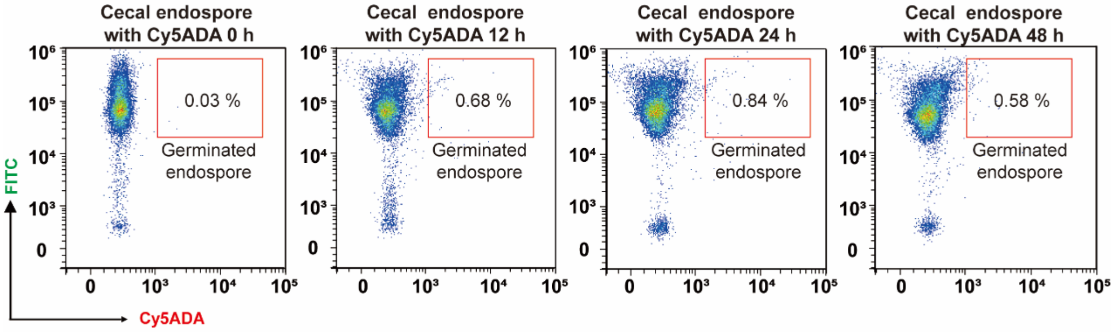

Figure S10. Flow cytometry-based evaluation of the spontaneous germination of cecal endospores during 48 h storage at 4°C in PBS.

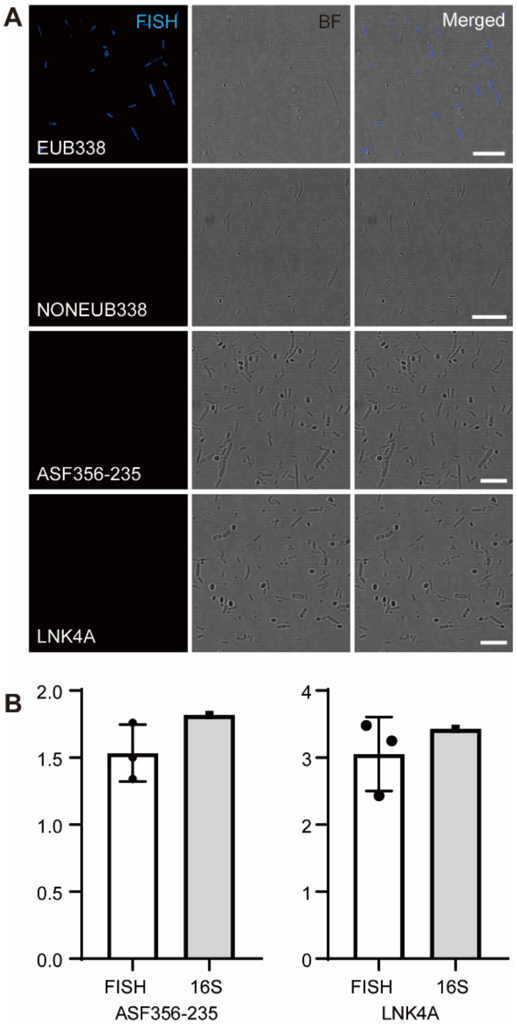

Figure S11. Staining specificities confirmation of the two new FISH probes. (**A**) Confocal fluorescence microscopy for assessing the specificity of the newly designed FISH probes against a soil microbiota sample. EUB338 and NONEUB338 probes were used as the positive and negative controls, respectively. Scale bars, 10 μm. (**B**) Comparison of the labeling ratios of the two probes against a gut microbiota sample with the relative abundances of the corresponding genera according to 16s rDNA sequencing. Three independent experiments were performed using FISH probes for *ASF356* and *Lachnospiraceae_NK4A136* to stain a mouse cecal microbiota which was also analyzed by 16s rDNA sequencing. The labeling ratios of each probe were analyzed by flow cytometry.

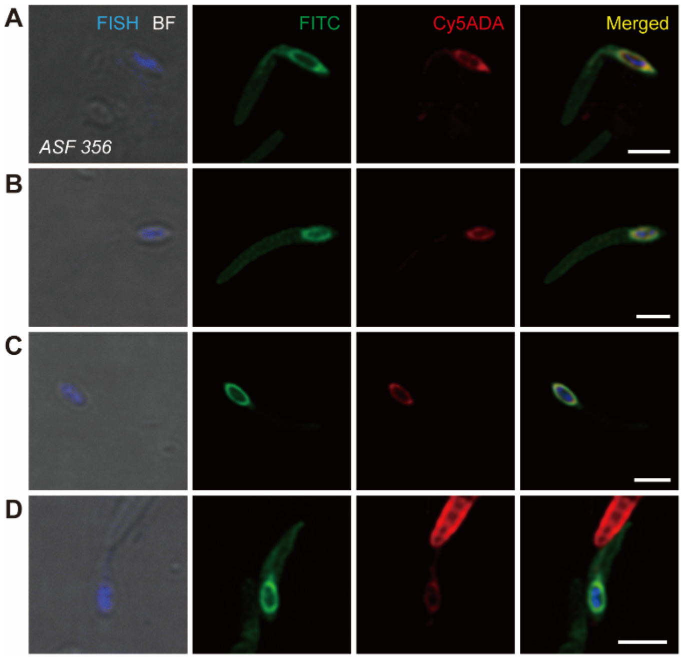

Figure S12. Confocal fluorescence imaging of the dually labeled and FISH-stained *ASF*356. The germinated cecal endospores which received two-step labeling of FITC (green) and Cy5ADA (red) were stained by ASF356-235 FISH probe (blue) targeting *ASF*356 at genus level. Representative images from three independent FISH staining experiments were shown. Scale bars, 2 µm.

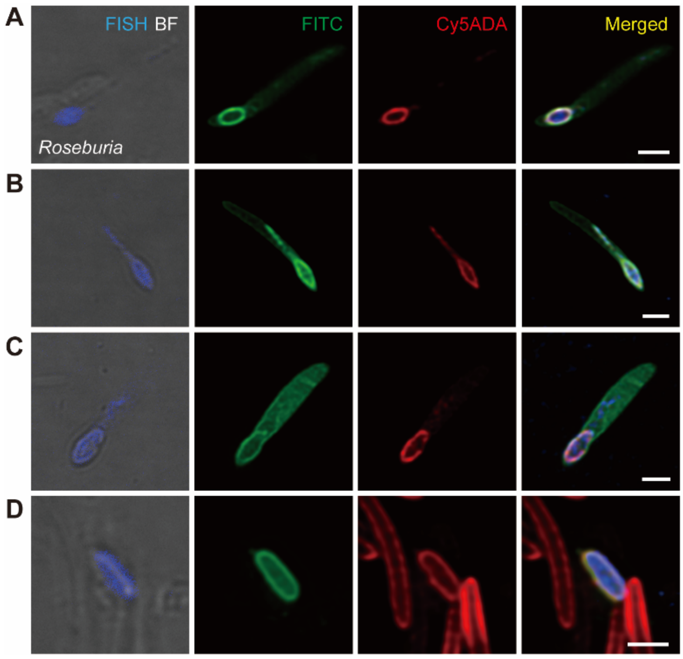

Figure S13. Confocal fluorescence imaging of dually labeled and FISH-stained *Roseburia***.** The germinated cecal endospores which received two-step labeling of FITC (green) and Cy5ADA (red) were stained by Rrec584 FISH probe (blue) targeting *Roseburia* at genus level. Representative images from three independent FISH staining experiments were shown. Scale bars, 2 µm.

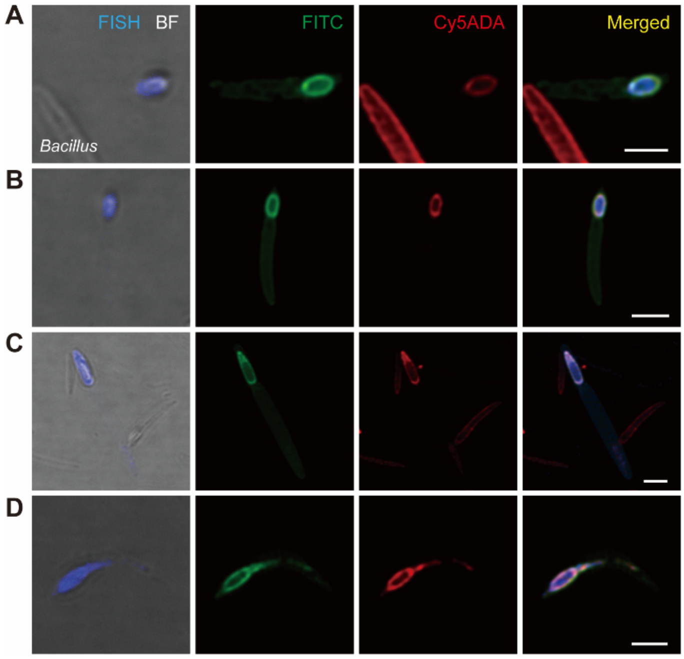

Figure S14. Confocal fluorescence imaging of dually labeled and FISH-stained *Bacillus*. The germinated cecal endospores which received two-step labeling of FITC (green) and Cy5ADA (red) were stained by Bmy843 FISH probe (blue) targeting *Bacillus* at genus level. Representative images from three independent FISH staining experiments were shown. Scale bars, 2 μm.

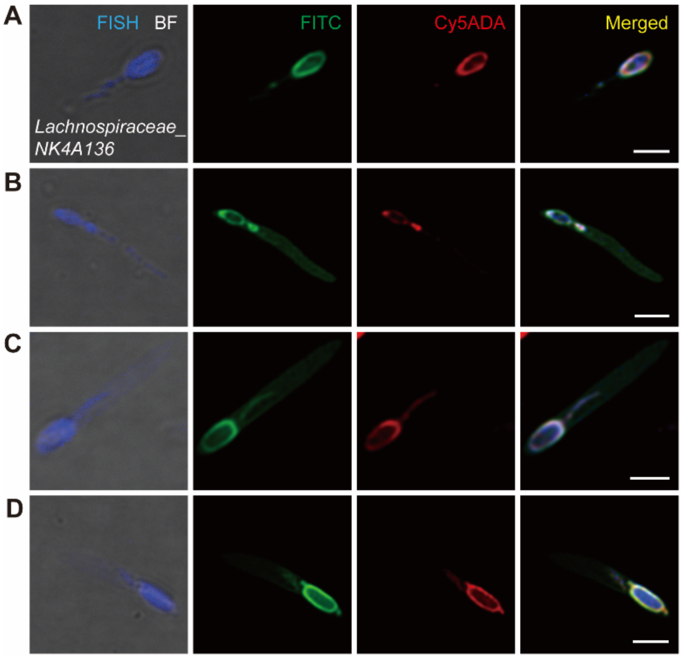

Figure S15. Confocal fluorescence imaging of dually labeled and FISH-stained *Lachnospiraceae_NK4A136*. The germinated cecal endospores received two-step labeling of FITC (green) and Cy5ADA (red) were stained by LNK4A FISH probe (blue) targeting *Lachnospiraceae_NK4A136* at genus level. Representative images from three independent FISH staining experiments were shown. Scale bars, 2 µm.

Supplementary Table S1. The FISH probes used in this study.

**Target Bacteria**		**Probe**	**Probe sequence （5’-3’）**	**Hybridization temp (°C)**	**Formamide Concn (%, v/v)**	**Source**
**Genus**	**Family of bacteria**					
Roseburia	*Lachnospiraceae*	Rrec584	TCA GAC TTG CCG YAC CGC	50	20	[49]
Bacillus	*Bacillaceae*	Bmy843	CTT CAG CAC TCA GGT TCG	46	0	[50]
Lachnospiraceae_ NK4A136	*Lachnospiraceae*	LNK4A	CACTGTTCCGAAGAAGGGTTCTGCA	46	20	This study
*ASF*356	*Clostridium spp.*	ASF356-235	CGGCGACTCCTCCCTCGCGGTTAGG	46	20	This study

**Target Bacteria**	**Probe**	**Probe sequence （5’-3’）**	**Hybridization temp (°C)**	**Formamide concn (%, v/v)**	**Source**
Most Bacteria	EUB338	GCT GCC TCC CGT AGG AGT	46	0	[51]
Control probe complementary to EUB338	NONEUB	ACT CCT ACG GGA GGC AGC	46	0	[52]
